# Improved YOLOv8-Based Target Precision Detection Algorithm for Train Wheel Tread Defects

**DOI:** 10.3390/s24113477

**Published:** 2024-05-28

**Authors:** Yu Wen, Xiaorong Gao, Lin Luo, Jinlong Li

**Affiliations:** School of Physical Science and Technology, Southwest Jiaotong University, Chengdu 610031, China; 2022201057@my.swjtu.edu.cn (Y.W.); happyluolin@vip.163.com (L.L.); jinlong_lee@126.com (J.L.)

**Keywords:** defect detection, neural network, target recognition, YOLOv8

## Abstract

Train wheels are crucial components for ensuring the safety of trains. The accurate and fast identification of wheel tread defects is necessary for the timely maintenance of wheels, which is essential for achieving the premise of conditional repair. Image-based detection methods are commonly used for detecting tread defects, but they still have issues with the misdetection of water stains and the leaking of small defects. In this paper, we address the challenges posed by the detection of wheel tread defects by proposing improvements to the YOLOv8 model. Firstly, the impact of water stains on tread defect detection is avoided by optimising the structure of the detection layer. Secondly, an improved SPPCSPC module is introduced to enhance the detection of small targets. Finally, the SIoU loss function is used to accelerate the convergence speed of the network, which ensures defect recognition accuracy with high operational efficiency. Validation was performed on the constructed tread defect dataset. The results demonstrate that the enhanced YOLOv8 model in this paper outperforms the original network and significantly improves the tread defect detection indexes. The average precision, accuracy, and recall reached 96.95%, 96.30%, and 95.31%.

## 1. Introduction

When it comes to train travel, safety is of utmost importance [1]. These defects can even pose a threat to the safety of high-speed train operations. External factors such as inertia, rail edge stress, and thermal damage can affect train wheels, leading to defects like bruises, peeling, and scratches on the tread surface. Ensuring the safety of high-speed train operations relies heavily on detecting wheel tread defects, but in the actual detection process, defects of various shapes and water stains often present challenges.

Conventional image detection methods include image localisation and segmentation, feature extraction, classifier classification, and so on. However, such methods are complex and have poor real-time performance, low efficiency, and difficulty meeting the needs of industrial production. Consequently, they have been gradually replaced by the use of deep learning to address these issues. The deep learning-based target detection method offers the advantages of fast detection speed and high accuracy, resulting in significant cost savings.

Deep learning-based target detection methods are typically classified into two types: region-based, two-stage detection algorithms, such as regions with CNNs (R-CNNs) [2], and faster region-based convolutional neural networks (Faster R-CNNs) [3]. The two-stage target detection network is transformed into a region proposal, followed by the utilisation of a neural network for target classification prediction and localisation implementation. Mengqi Chen et al. [4] utilised the Gabor filter in frequency analysis and integrated the Gabor kernel into the Faster R-NN model to address fabric detection with background texture interference. Xiangyang Xu et al. [5] conducted a comparative analysis of Faster R-CNN and Mask R-CNN for road crack detection.

The you-only-look-once (YOLO) [6] series of one-step detection algorithms represents another category. The one-stage target detection network directly extracts features and classifies and locates targets through the neural network, resulting in improved efficiency compared to the two-stage target detection network. Abhiroop Bhattacharya et al. [7] used a combination of current techniques in deep learning-based transformers, multilevel feature fusion, data augmentation, and object detection to quickly detect PCB board defects. Stefano Frizzo Stefenon et al. [8] used a genetic algorithm to optimise the hyperparameters of the YOLO model for grid fault localisation, successfully improving performance metrics and reducing computational requirements. Fenglong Ding et al. [9] employed transfer learning to apply a single-shot multibox detector (SSD) for wooden panel defect detection, utilising DenseNet as the backbone network instead of VGG16 and incorporating residual learning to mitigate feature information loss, thereby achieving wood defect detection. Qiqi Zhou et al. [10] proposed CABF-YOLO, an accurate and efficient strip surface defect detection model that incorporates a novel Bidirectional Fusion (BF) strategy to acquire detailed information and cater to the requirements of industrial strip surface defect detection.

YOLO series algorithms are widely employed for the purpose of defect detection in various applications [11]. However, the detection performance of YOLOv8 may not always meet the requirements for detecting special scenes. Defect detection in tread defect images is susceptible to variations in lighting conditions and water stains, while the intricate background of the tread image poses interference to accurate defect detection. To address this issue, this paper proposes an enhanced version of YOLOv8 that improves upon the original model by enhancing wheel tread defect detection.

1. In order to solve the problem of the false detection of water stains in defects, this paper optimises the network detection structure by adding a shallow small-target detection layer and deleting the large-target detection layer, which not only attenuates the impact of water stains but also pays more attention to the detection of small target defects.
2. To prevent the location information of small targets from being overlooked during the learning process, this paper uses an improved SPPCSPC (Spatial Pyramid Pooling Concurrent Spatial Pyramid Convolution). Firstly, the CBS (Conv BatchNormal Silu) is trimmed to address the issue of filtering the edge information of small target defects, such as peeling. Then, the SimAM (simple attention mechanism) is introduced to minimise loss in the feature extraction of small target defects.
3. This paper replaces the loss function with SIOU (Soft IoU), which encompasses four key components: angle loss, distance loss, shape loss, and IoU loss. This modified approach offers a more comprehensive solution compared to the original CIOU (Complete IoU), as it takes into account multiple factors, resulting in improved convergence speed and higher accuracy.

## 2. YOLOv8 Network Structure

**Figure 1:**
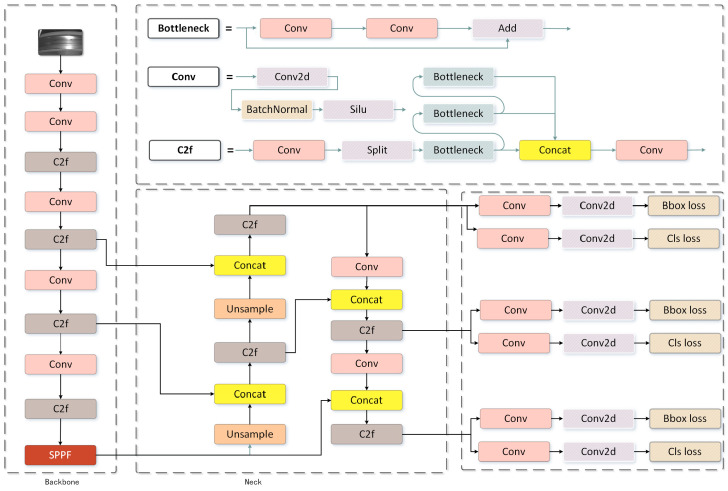
The structure of the YOLOv8 module.

The YOLOv8 algorithm provides a new SOTA (state of the art) model that can be used for tasks such as target detection [12], image segmentation [13], etc., and its main structure consists of three parts: the backbone, the neck, and the head. The complete network structure of YOLOv8 is illustrated in Figure 1.

The C2f module based on the CSP is used in the main part instead of the C3 module. C2f utilises the concept of ELAN in YOLOv7 and combines C3 with ELAN. This enables YOLOv8 to maintain high computational efficiency and obtain more information about the gradient flow simultaneously. The last pyramid space pooling is replaced with the SPPF module, which can extract spatial information more effectively.

In the neck section, YOLOv8 continues to use the PAN-FPN (Path Aggregation Network with Feature Pyramid Network) feature fusion technique, which effectively combines information from feature layers at various scales. It employs multiple up-sampling procedures and C2f modules, as well as a final decoupled head structure, to create the neck module. The confidence and regression frames are combined to achieve greater accuracy.

In the head section, YOLOv8 employs an anchorless split detection head, thereby enhancing both detection accuracy and efficiency in comparison to the anchor frame-based method. The head structure is replaced with the mainstream decoupled head structure to alleviate the conflict between classification and localisation tasks. The reference is changed from anchor-based to anchor-free for YOLOX. Furthermore, YOLOX has been updated from an anchor-based to an anchor-free approach, which is particularly beneficial for detecting faces with irregular dimensions.

## 3. Improved YOLOv8 Structure

Although YOLOv8 has performed well in all aspects of the detection task, it still faces difficulties in recognising confusing objects. During feature extraction for defects on the tread surface, the neural network may mistake water stains for defects due to their similarity in image information. Additionally, YOLOv8 may miss small defects on the tread surface.

To address the aforementioned issues, this paper proposes a defect detection algorithm that can effectively identify small defects on the tread surface while avoiding the misdetection of water stains. Firstly, we optimised the large detection head in the original network to increase the structure of the small detection head, thereby reducing misdetection caused by water stains. Secondly, we replaced the original SPPF with the SPPCSPC module, which was optimised and introduced into the SimAM to prevent the misdetection of small defects. Throughout the learning process, the algorithm does not ignore the target’s position information. Finally, the loss function is replaced with SIOU, which considers more geometric factors and improves the accuracy of detecting tread defects.

### 3.1. Optimisation of Network Structure

As water stains and defects share similar visual features, it is easy to mistake water stains for defects. In order to show the response of water stains and defects in different network layers for subsequent reduction of false detection of water stains. In this paper, GradCAM [14] is used to generate heat maps, based on which we can better analyse the regions of interest of the neural network.

**Figure 2:**
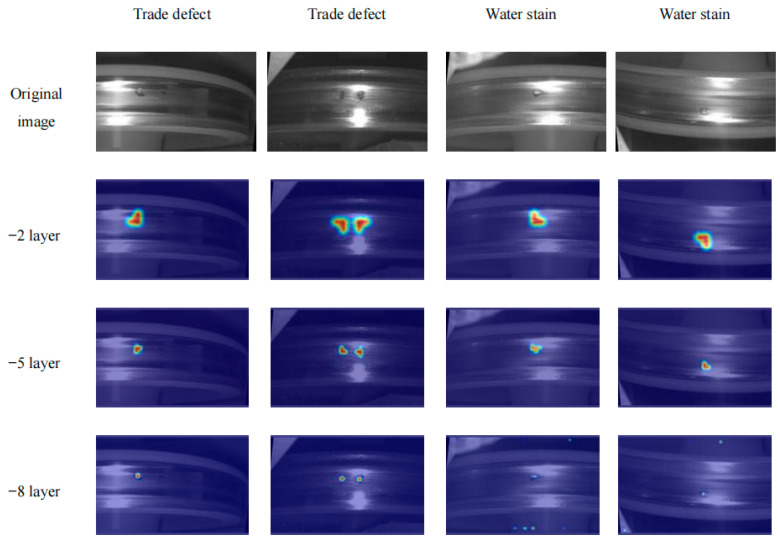
Tread thermal response diagram.

Figure 2 integrates a heat map of water stains and defects. Upon analysis of the figure, it becomes apparent that the deep layer of the network employed for large-target detection exhibits a relatively diminished response disparity between defects and water stains. Conversely, the shallow layer of the network utilised for detecting small targets demonstrates enhanced efficacy in filtering out water stains on the tread surface. Consequently, by eliminating the large-target detection layer and incorporating a small-target detection layer, the final detection outcomes are capable of mitigating the impact of water stains, thereby enabling improved focus on identifying small target defects.

**Figure 3:**
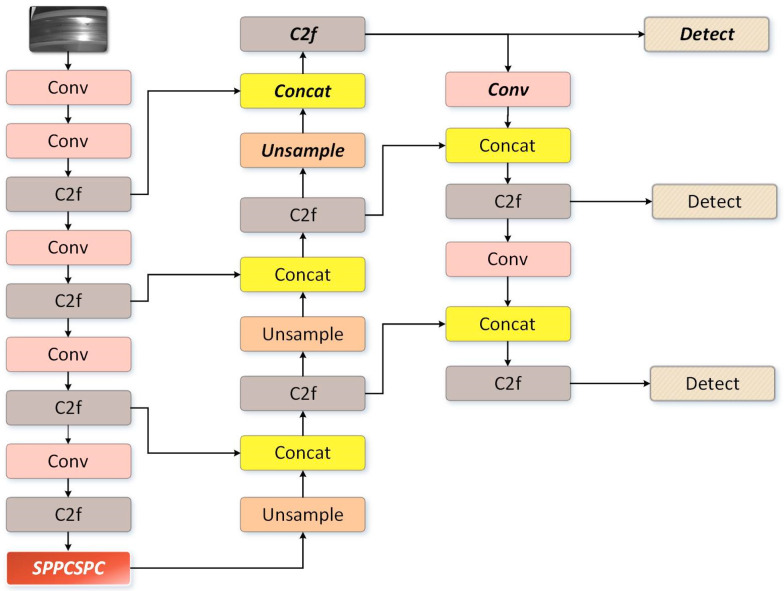
The structure of the improved YOLOv8 module.

To address the problem of the misidentification of water stains in the original algorithm, the original YOLOv8 generates three feature maps of different sizes. In this study, we removed the 20 pixel × 20 pixel large-target detection layer and added a 160 pixel × 160 pixel small-target detection layer to improve defect detection. The architecture of the improved YOLOv8s network detection layer is shown in Figure 3. Although YOLOv8 achieves a larger perceptual domain through deep sampling, deep sampling results in the loss of some feature information. By incorporating a shallow network, we can better extract detailed features of the target, preserve defect-related information, reduce the interference of water spots on defect detection, mitigate the loss of small target defect features, and improve the small-target detection capability. The improved network exhibits reduced sensitivity to water stains and provides more accurate defect detection results.

### 3.2. Reconstructed SPPCSPC

**Figure 4:**
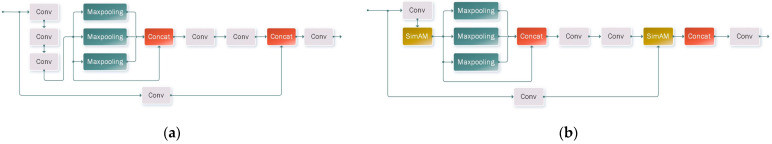
SPPCSPC structure diagrams: (**a**) SPPCSPC before reconstruction; (**b**) SPPCSPC after reconstruction.

The original network exhibits limited capability for detecting small defects in the wheel tread. To enhance its defect detection ability and accurately locate defects, we replace the initial SPPF module with an improved SPPCSPC module. This novel SPPCSPC module integrates spatial pyramid pooling (SPP) [15] and a cross-stage Partial Channel (CSPC) [16], surpassing the performance of the SPPC alone. By employing serial computation, the SPPCSPC module adapts to images of varying resolutions while accelerating the convergence speed. To better suit the spatial pyramid pooling of tread features, we reconstruct the SPPCSPC module as depicted in Figure 4a,b.

Firstly, the two CBS layers located before the pooling layer are cropped out to reduce the filtering of small-target edge information by the convolutional layer. This also scales down the computational load of the neural network. Secondly, the SimAM [17] attention mechanism is introduced to address the issue of the inadequate extraction of small, defective target features. SimAM assigns a 3D attention weight to each neuron while dividing the pixels between the complex background region and the small-target region to enhance the sensitivity of the complex background and small-target regions. Adding SimAM before the pooling layer and final channel merging can help divide the defective and non-defective regions, enhancing the network’s attention to the small-target region, reducing feature loss, and improving the model’s robustness by reducing the influence of the tread background on the defects.

### 3.3. SIoU

The purpose of this function is to continuously optimise the fitting effect by defining the calculation method around the geometrical elements of the prediction and real frames. Representative methods include GloU [18], CloU [19], and EloU [20]. The regression loss function of YOLOv8s adopts the CloU loss function, which considers the overlap area, distance from the centroids, and aspect ratio. Although CloU considers more comprehensive factors compared to the previous loss function, it does not take into account the mismatch of directions between the real and predicted frames. To improve the model’s generalisation ability and accelerate convergence, the network model’s border loss function is replaced by SloU from CloU [21]. SIoU considers the vector angles between regressions and redefines the penalty indicator. The SloU loss function converges faster and is more accurate than the CIoU loss function. The SIoU loss function comprises angular loss, distance loss, shape loss, and IoU loss.

**Figure 5:**
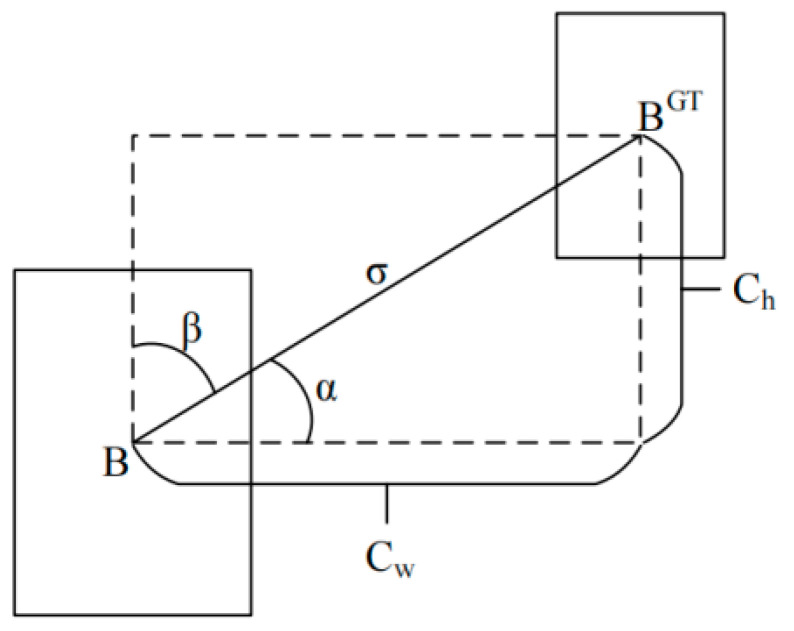
Calculation of angle loss.

Figure 5 shows the angular loss map of SIOU, where σ represents the distance between the centre point of the real frame and the predicted frame, $C_{h}\;$ represents the height difference between the centre point of the real frame and the predicted frame, α and represents the horizontal angle between the predicted frame and the real frame.

The regression direction of the prediction frame is determined by the angular magnitude, where the angular loss is defined as follows:

(1) $$\Lambda =1-2{sin}^{2}(arcsin(x)-\frac{\pi }{4})$$

(2) $$\left\{\begin{array}{c} x=\frac{C_{h}}{\sigma }=\sin(\alpha )\\ \sigma =\sqrt{(b_{c_{x}}^{gt}-b_{c_{x}}{)}^{2}+(b_{c_{y}}^{gt}-b_{c_{y}}{)}^{2}}\\ C_{h}=\max(b_{c_{y}}^{gt},b_{c_{y}})-\min(b_{c_{y}}^{gt},b_{c_{y}}) \end{array}\right.$$

where σ is the distance between the centre point of the real frame and the predicted frame, and α is the horizontal angle between the predicted frame and the real frame

**Figure 6:**
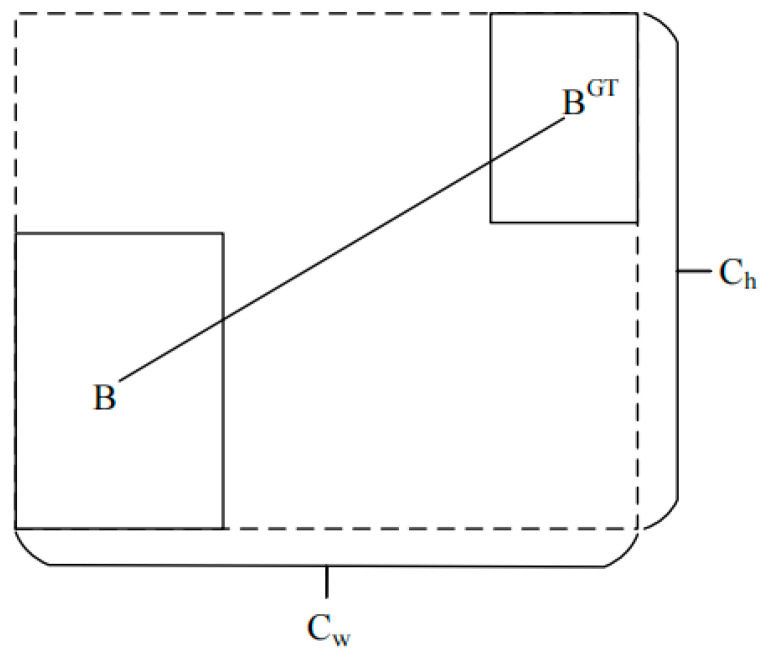
Calculation of distance loss.

The calculation diagram for distance loss is shown in Figure 6.

The distance loss is defined as follows:

(3) $$\Delta =\sum_{t=x,y}\left(1-e^{-\gamma \rho_{t}}\right)$$

(4) $$\left\{\begin{array}{c} \rho_{x}=(\frac{b_{c_{x}}^{gt}-b_{c_{x}}}{c_{w}}{)}^{2}\\ \rho_{y}=(\frac{b_{c_{y}}^{gt}-b_{c_{y}}}{c_{h}}{)}^{2}\\ \gamma =2-\Lambda \end{array}\right.$$

In the formula for distance loss, ρ is the degree of positional deviation between the true and predicted frames, and γ is the preferred distance value for time, related to angular loss. It can be seen that the contribution of the distance cost is greatly reduced when α → 0. On the contrary, the closer it is to π/4, the greater the contribution of the distance cost.

Shape loss is defined as follows:

(5) $$\Omega =\sum_{t=w,h}(1-e^{-w_{t}}{)}^{\theta }$$

(6) $$\left\{\begin{array}{c} \omega_{w}=\frac{\left|w-w^{gt}\right|}{max(w,w^{gt})}\\ \omega_{h}=\frac{\left|h-h^{gt}\right|}{max(h,h^{gt})} \end{array}\right.$$

where θ is the degree of concern for shape loss, $\omega_{w}$ and $\omega_{h}$ are the degrees of stretching of the width and height between the predicted and real frames, and $w^{gt}$ and $h^{gt}$ are the width and height of the real frame.

IoU cost is defined as follows:

(7) $$IoU=\frac{\left|A\cap B\right|}{\left|A\cup B\right|}$$

where A denotes the prediction frame and B denotes the true frame.

SIoU is defined as follows:

(8) $$L_{SIoU}=1-L_{IoU}+\frac{∆+\Omega }{2}$$

## 4. Experimental Section

### 4.1. Dataset and Metrics

The dataset comprises 6547 images of tyre treads captured by the camera, where image quality is affected by light brightness at the time of capture, and background interference outside the tread area hinders defect detection. For small defects, noise and light interference pose challenges for feature extraction by the network. Additionally, water stains on the surface can lead to misclassification as defects.

**Figure 7:**
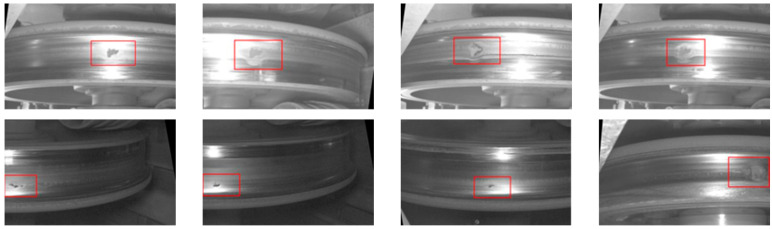
Sample images of tread defects.

Sample images of tread defects are shown in Figure 7 with tread defects in the red box, in which there are lighting differences and water stains distributed on the tread, and the morphology of the defects is also different, which affects the recognition of defects on the tread. The tread data are partitioned into training, validation, and test sets in a ratio of 7:2:1, with the classified image datasets being organised into their respective directories to facilitate subsequent model training.

The network’s training parameters were configured according to Table 1, utilizing the Adma optimizer with a Momentum value of 0.8, Learning Rate set at 0.01, Batch Size of 16, Epochs set to 100, and Weight Decay adjusted to 0.0005. The experimental platform has a GPU of RTX3060 (NVIDIA, Santa Clara, CA, USA) and a CPU of 12th Gen Intel^®^ Core™ i7-12700H (Intel, Santa Clara, CA, USA).

Precision (P), recall (R), and mean average precision (mAP) values for all categories were selected as evaluation criteria and calculated as follows:

(9) $$P=\frac{TP}{TP+FP}$$

(10) $$R=\frac{TP}{TP+FN}$$

(11) $$mAP=\frac{\sum_{i1}^{N}AP_{i}}{N}AP=\int_{0}^{1}P(R)dR$$

(12) $$\mathrm{mAP}@50\%=\frac{1}{C}\sum_{i=1}^{C}AP@0.5_{i}$$

### 4.2. Ablation Experiment

In order to verify the effect of the improved algorithm on YOLOv8, ablation experiments were conducted, and the results are shown in Table 2. And model parameters and FPS were added as evaluation indicators.

The table uses a checkmark symbol (√) to indicate the corresponding improvement strategy. The same training parameters are applied to each group of experiments. Optimisation model 1 improves the detection structure by replacing the large target detection head with a shallow small-target detection head that is more sensitive to small defects. This reduces the misjudgement of water stains and improves detection accuracy. The accuracy of the detection results is improved by 6.75% and the mAP by 6.16% compared to the original model. 

Optimisation model 2 replaces the original SPPC module with the SPPCSPC module, optimises its structure, trims the original CBS, and introduces SimAM. The multiscale structure of SPPCSPC, the sensory field of the neural network, and the small targets are now better matched, resulting in improved detection accuracy for the model. However, the number of parameters has slightly increased. 

Optimisation model 3 uses SloU as the loss function for border regression, resulting in more effective optimisation and improved positioning precision for the model bounding box. 

Compared to the original YOLOv8, the improved version achieves a detection accuracy of 96.30%, which is 9.84% higher than the base model; a recall rate of 95.31%, which is 11.95% higher than the previous result; and an mAP of 96.95%, an improvement of 8.76%. The original YOLOv8 profile has a Missing Alarm Rate (MAR) of 16.64%. The improved YOLOv8 network reduced the false alarm rate for tread defects to 4.69%, a reduction of 11.95%.

**Figure 8:**
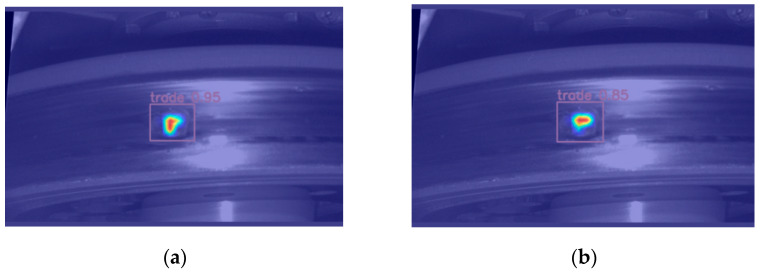
Grad-CAM visualisation: (**a**) improved YOLOv8; (**b**) YOLOv8.

Figure 8 shows the visualisation of the tread defect features of YOLOv8 and the improved YOLOv8, which can more clearly show the feature extraction by the network. From the figure, we can see that the improved YOLOv8 network detects defects better and with higher confidence.

**Figure 9:**
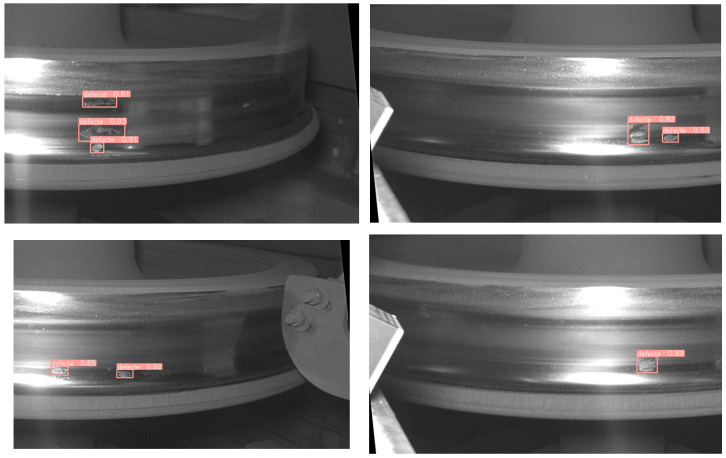
Results of the improved Yolov8 algorithm in small tread defect detection.

The improved YOLO8 algorithm is demonstrated in Figure 9, showcasing its proficiency in detecting minute defects on the wheel’s tread surface. This improved neural network exhibits remarkable capability in identifying small defects present on the tread.

### 4.3. Method Comparison Experiment

In order to further verify the effectiveness of the improved algorithm, we compare the enhanced yolov8 in this paper with several mainstream target detection algorithms on the treadmill dataset, and the results are shown in Table 3. The evaluation metrics of the model include accuracy, recall, MAP and model parameters.

The metrics of this paper’s model and other commonly used models for tread detection are presented in Table 3. The mAP score achieved by our proposed model surpasses that of existing algorithms, reaching an impressive 96.95%, thus demonstrating its superior performance in comparison. Notably, accurately detecting defects in the tread under challenging conditions such as varying lighting and water stains has been a persistent challenge for existing methods. However, the improved YOLOv8 algorithm proposed in this study effectively addresses these challenges.

YOLOv8 builds upon the earlier versions (YOLOv3 and YOLOv5) by incorporating advancements like the C2F module and decoupled head while abandoning the anchor-based concept in favour of an anchor-free approach. On the other hand, the SSD model suffers from a loss of target feature information due to multilayer convolution, making it less effective at detecting small targets. Faster R-CNN solves the problem of scale variation through its pyramid model, but still suffers from computational redundancy in the subsequent detection phase.

Compared with Faster R-CNN and YOLOv5s, our method showcases improvements of 10.71% and 11.5% in terms of accuracy, respectively, along with an increase in the mAP of 13.12% and 17.65%.

**Figure 10:**
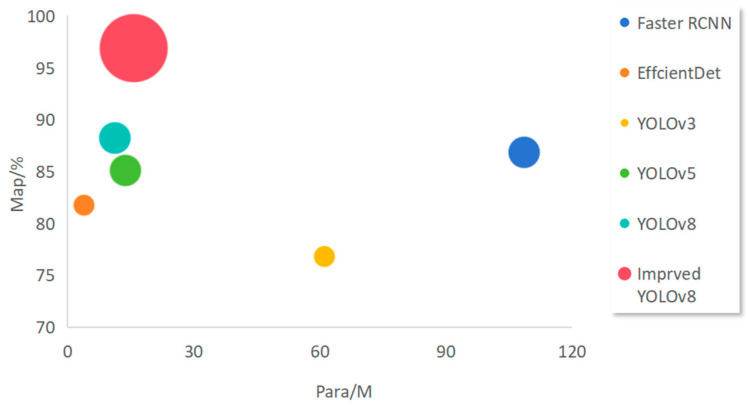
Comparison results with mainstream algorithms.

A comparison of our model with other models in terms of parameters and accuracy is visualised in Figure 10, where the horizontal axis represents the parameters of the model and the vertical axis represents the MAP. It can be observed that our model exhibits superior accuracy while maintaining a lower parameter count.

## 5. Conclusions

This paper proposes an improved YOLOv8 algorithm for detecting high-speed train wheelset treads. To avoid interference from water stains during defect detection, the detection structure in the YOLOv8 model has been improved by adding a small-target, shallow detection layer. Furthermore, the SPPC module has been replaced with the reconstructed SPPCSPC to achieve a more efficient multiscale fusion that prioritises small-target defects. Additionally, the loss function has been substituted with SIoU to enhance the accuracy of the regression. The algorithm proposed in this study was compared to current target detection algorithms on a self-built dataset to evaluate its wheel-to-tread detection performance. The results indicate that the proposed algorithm outperforms the original YOLOv8 model, achieving a detection rate of 96.95% for tread defects, which is an improvement of 8.76%. In the future, we will pay more attention to the improvement of detection speed in real time and further improve the accuracy of detection.

## Figures and Tables

**Table 1:** Experimental training parameters.

Training Parameters	Value
Optimiser	Adam
Momentum	0.8
Learning Rate	0.01
Batch Size	16
Epoch	100
Weight Decay	0.0005

**Table 2:** Results of ablation experiments.

Model	Structural Optimisations	SPPCSPC	SIoU	Precision	Recall	mAP@0.5	Parameters	FPS
YOLOv8				86.46	83.36	88.19	11.4	89
Optimisation models 1	√			93.21	90.66	94.35	12.8	86
Optimisation models 2		√		90.96	88.67	91.87	18.2	85
Optimisation models 3			√	94.05	89.06	92.92	11.5	88
Ours	√	√	√	96.30	95.31	96.95	18.9	79

**Table 3:** Comparison results of tread defect detection.

Model	Precision	Recall	mAP@0.5	Parameters
Faster R-CNN	80.38	82.03	83.70	40.2
SSD [22]	47.02	88.76	78.12	26.3
YOLOv3 [23]	75.59	72.19	76.80	61.5
YOLOv5s	80.12	84.19	85.15	7.1
YOLOv8s	86.46	83.36	88.19	11.4
Improved YOLOv8	96.30	95.31	96.95	18.9

## Data Availability

Data are contained within the article.
